# MARCH2 inhibits avian leukosis virus replication by targeting gp85 for ubiquitination and degradation

**DOI:** 10.1128/jvi.01616-25

**Published:** 2025-12-03

**Authors:** Yuntong Chen, Longbo Wu, Wenrui Fan, Ying Wang, Jinglei Li, Xuefu Zhang, Zibo Zhang, Yumeng Li, Suyan Wang, Yongzhen Liu, Xiaole Qi, Yanping Zhang, Hongyu Cui, Yulu Duan, Yulong Gao

**Affiliations:** 1 Avian Immunosuppressive Diseases Division, State Key Laboratory for Animal Disease Control and Prevention, Harbin Veterinary Research Institute, The Chinese Academy of Agricultural Sciences 111613 , Harbin, China; 2 Jiangsu Co-innovation Center for Prevention and Control of Important Animal Infectious Disease and Zoonoses, Yangzhou, China; Icahn School of Medicine at Mount Sinai, New York, New York, USA

**Keywords:** ubiquitination, degradation, gp85, MARCH2, avian leukosis virus

## Abstract

The replication of avian leukosis virus (ALV), a simple retrovirus that induces neoplastic diseases in chickens, is regulated by host factors. Investigating the antiviral activity of these host factors and the underlying mechanisms could provide a theoretical foundation for the prevention and control of ALV infections. In this study, RNA sequencing revealed that ALV-A infection upregulates the expression of membrane-associated RING-CH 2 (MARCH2). Increasing MARCH2 levels inhibited ALV-A replication by 3.47- to 16.98-fold in viral titer, while conversely, knockout of MARCH2 promoted it by 5.04- to 10.70-fold. MARCH2 was found to interact with the viral gp85 protein, catalyzing its K27-linked ubiquitination and proteasomal degradation at lysine 282 (K282). Substituting K282 with arginine prevented degradation and enhanced ALV-A replication. Importantly, the K282 site was determined to be conserved across various ALV subgroups, with its mutation disrupting MARCH2-mediated degradation. Our findings not only identified MARCH2 as a robust defense mechanism against multiple ALV subgroups, functioning by targeting viral proteins, but also provide a potential target for novel strategies against avian leukosis.

**IMPORTANCE:**

Avian leukosis virus (ALV), a simple retrovirus, not only causes chicken tumor disease and immunosuppression but, more importantly, can spread vertically through hatching eggs, affecting the quality of chicks and endangering the safety of poultry breeds. Currently, no vaccines or treatments are available, and the most effective strategy for preventing and controlling this disease in chicken flocks is the eradication of ALV. The MARCH protein family exhibits antiviral activity in mammals. Our research found that the MARCH protein family has antiviral functions in birds. Further, MARCH2 was determined to bind gp85 in different subgroups of ALVs, ubiquitinate and facilitate the degradation of gp85 via the K282 residue, and inhibit the replication of different ALV subgroups. This study significantly advances our understanding of the avian defense mechanisms against viral infections and offers new targets for developing novel ALV prevention and control strategies.

## INTRODUCTION

Avian leukosis, a neoplastic disease caused by the avian leukosis virus (ALV), results in tumor development, reduced fertility, and stunted growth. During ALV replication, its genome is integrated into chicken chromosomes, leading to lifelong carriage and vertical transmission to offspring (1–4). However, despite extensive research, no effective vaccines or therapeutics have been developed to combat this condition. The primary strategy for controlling ALV is to eradicate the infected breeder flocks. ALV is divided into 11 subgroups based on cross-neutralization, its host range, and envelope interference (5, 6). Further, it has a 7.3 kb RNA genome encoding some essential proteins, such as envelope glycoproteins (gp85) (7–12). Moreover, owing to its reliance on host factors for replication, novel prevention and control strategies targeting host factors are urgently needed.

ALV infections represent an ongoing battle with the host, wherein the virus exploits both viral and host factors to facilitate replication, whereas the host counteracts this by inducing antiviral responses to combat the infection (13). The expression levels of several host factors, such as the tripartite motif-containing proteins TRIM25 (14, 15) and TRIM62 (16, 17), are upregulated during ALV infection and can restrict viral replication. Moreover, the tumor suppressor protein p53 inhibits ALV-J transcription by interacting with the viral long terminal repeat, thereby affecting its replication (18, 19). Long-chain acyl-CoA synthetase 1 (ACSL1), an interferon (IFN)-stimulated gene, specifically restricts the replication of ALV-J due to its role in enhancing IFN-I production (20). Accordingly, investigating the molecular mechanisms through which host factors restrict ALV replication, such as upregulation of the expression levels of these antiviral host factors in chickens, will contribute to a deeper understanding of novel ALV infection mechanisms and aid in the development of new prevention and control strategies to ensure the diversity of chicken breeds.

The MARCH family of membrane-associated RING-CH (MARCH) proteins, comprising E3 ubiquitin (Ub) ligases that are distinguished by their conserved N-terminal RING-CH domain, exert pivotal effects on the stability, trafficking, and functionality of many cellular membrane proteins (21–25). These proteins are instrumental in curtailing viral replication and mitigating the severity of viral pathogenesis. Notably, MARCH8 impedes HIV-1 replication by downregulating viral envelope glycoprotein expression, thereby preventing its incorporation into virions (26–28). In contrast, MARCH8 not only suppresses the presence of VSV-G on the cell surface but also facilitates its degradation within lysosomes (29). Moreover, MARCH2, MARCH8, and MARCH9 facilitate the polyubiquitination of furin, thereby inhibiting its enzymatic activity, which is crucial for the maturation of enveloped viral proteins, and this thereby potentially disrupts the viral life cycle (30). Conversely, certain viruses, including hepatitis C, dengue, and Zika virus, exploit MARCH8 to ubiquitinate viral proteins, thereby enhancing viral replication (31). This duality underscores the intricate interplay between MARCH proteins and different viruses, highlighting the need for a nuanced understanding of their interactions.

In this study, we explored the effect of various MARCH proteins on ALV replication and found that MARCH2 expression is upregulated in response to ALV infection and significantly suppresses viral replication. Our findings indicate that MARCH2 interacts with ALV gp85, leading to its ubiquitination and subsequent proteasomal degradation, thereby curtailing ALV replication. This study revealed a host-restrictive mechanism targeting ALV infection and elucidated the regulatory pathway through which MARCH2 inhibits ALV replication, thereby providing new targets for the development of novel ALV prevention and control strategies.

## RESULTS

### ALV-A infection increases *MARCH2* expression *in vitro*

**Fig 1:**
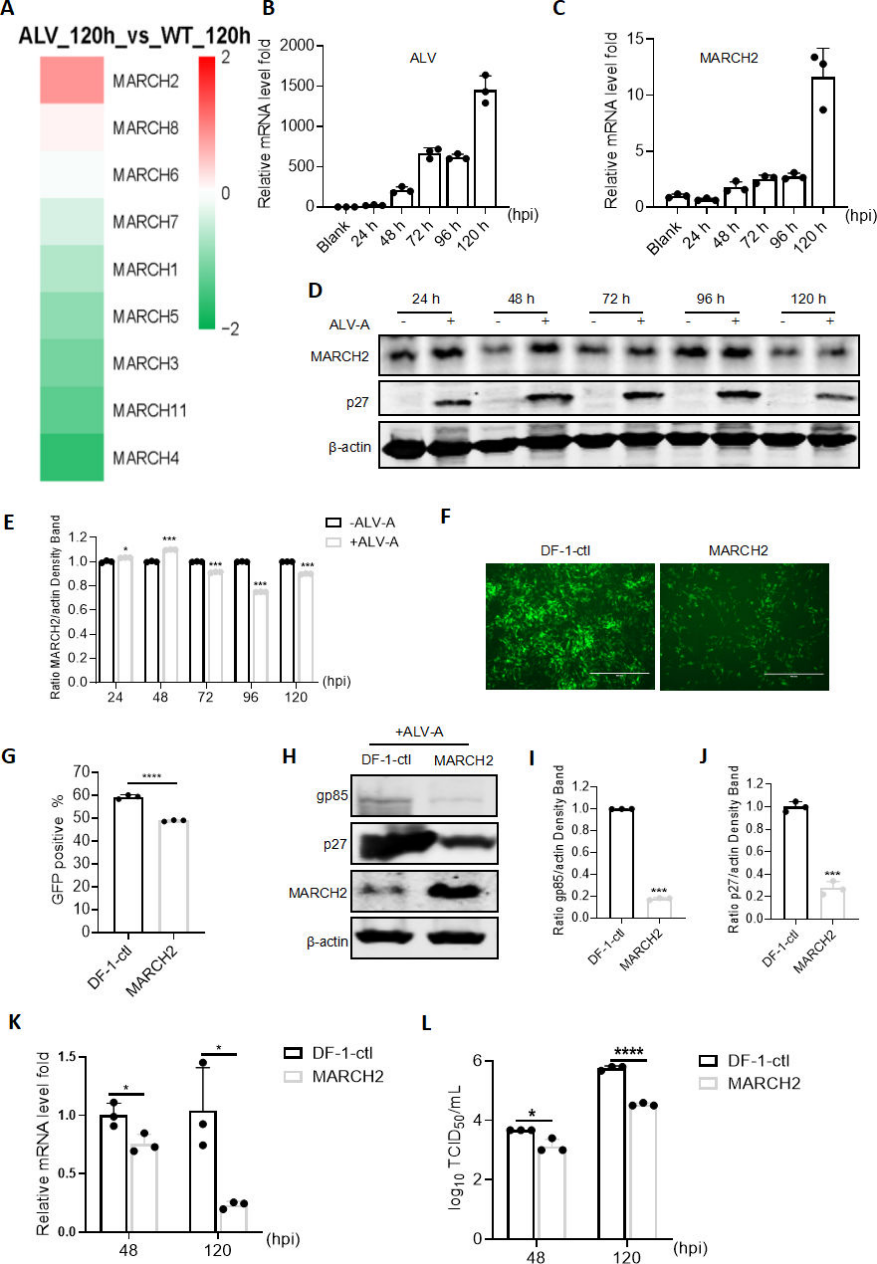
MARCH2 overexpression inhibits ALV-A replication. (**A**) Results of RNA-sequencing analysis of DF-1 cells challenged with ALV-A (multiplicity of infection [MOI] = 0.1) at 120 h post-infection (hpi). (**B and C**) The transcriptional upregulation of MARCH2 expression in DF-1 cells upon ALV-A infection is demonstrated. DF-1 cells were either inoculated with ALV-A (MOI = 0.1) or left untreated and then harvested at 24, 48, 72, 96, and 120 hpi. (**B**) Quantification of ALV-A genomic copies *in vitro* via RT-qPCR. (**C**) Assessment of *MARCH2* relative expression levels via RT-qPCR. (**D**) The expression of MARCH2 in DF-1 cells upon ALV-A infection is demonstrated via western blotting. DF-1 cells were either inoculated with ALV-A (MOI = 0.1) or left untreated and then harvested at 24, 48, 72, 96, and 120 hpi. (**E**) Relative intensities of MARCH2 were normalized with β-actin in the cell lysis. (**F and G**) Effect of MARCH2 overexpression on ALV-A-GFP replication. (**F**) Viral infectivity was assessed under a fluorescence microscope following exposure to ALV-A-GFP for 120 h. Scale bar = 125 µm. (**G**) Determination of viral infectivity via flow cytometry, quantifying the percentage of green fluorescent protein (GFP)-positive cells post-ALV-A-GFP infection for 120 h. (**H–L**) Effect of MARCH2 overexpression on wild-type ALV-A replication. (**H**) Quantification of p27 and gp85 protein expression via western blotting post-ALV-A infection for 120 h. (**I**) Relative intensities of gp85 were normalized with β-actin in the cell lysis. (**J**) Relative intensities of p27 were normalized with β-actin in the cell lysis. (**K**) Calculation of the relative fold change in ALV-A genomic mRNA levels in cell lysates via RT-qPCR post-ALV-A infection for 48 or 120 h. (**L**) Determination and visualization of the released viral titer, based on the tissue culture infectious dose (TCID_50_) assay post-ALV-A infection for 48 or 120 h. *P < 0.05, **P < 0.01, and ****P < 0.0001.

To elucidate the dynamic expression patterns of various MARCH family members in response to ALV-A infection, an RNA-Seq experiment was performed. Among several MARCH family proteins that exhibit changes in expression levels, most MARCH family genes showed downregulated expression. In particular, *MARCH4* was highly downregulated, while *MARCH2* expression was the most significantly upregulated. The significant upregulation of *MARCH2* expression suggests that it may be a key regulatory factor in the ALV-A replication cycle (Fig. 1A). To explore the relationship between *MARCH2* expression and ALV infection, DF-1 cells were infected with ALV-A (RAV-1 strain) at a multiplicity of infection (MOI) of 0.1, and changes in *MARCH2* mRNA and protein expression levels were detected. The transcription levels of the ALV *gag* gene within the cells and intrinsic *MARCH2* expression were measured using quantitative reverse transcription-PCR (RT-qPCR) at 24, 48, 72, 96, and 120 h post-infection. RT-qPCR results confirmed the establishment of ALV-A infection, with the relative mRNA levels of ALV-A genes peaking at a 1,500-fold increase in DF-1 cells (Fig. 1B). In parallel, *MARCH2* expression was notably enhanced at multiple time points compared to that in non-infected cells, culminating in a 10-fold increase in ALV-A-infected DF-1 cells at 120 h post-infection (Fig. 1C). Western blotting results showed that the expression level of *MARCH2* increased with ALV-A infection at 48 h post-infection (Fig. 1D and E). In summary, these results suggest that ALV-A infection triggers the upregulation of *MARCH2* expression *in vitro*.

### MARCH2 inhibits ALV-A replication *in vitro*

To elucidate the regulatory role of MARCH2 in ALV infection, a DF-1 cell line overexpressing *MARCH2* (DF-1-MARCH2) was engineered. Western blotting analysis confirmed that the expression level of *MARCH2* was significantly higher in the DF-1-MARCH2 cell line compared to that in control DF-1 cells (DF-1-ctl) (Fig. S1). Subsequently, to ascertain the effect of *MARCH2* overexpression on ALV-A replication, DF-1-ctl and DF-1-MARCH2 cells were infected with an ALV-A strain expressing green fluorescent protein (GFP) (ALV-A-GFP). Fluorescence observations indicated that *MARCH2* overexpression reduced ALV-A-GFP replication (Fig. 1F), and a flow cytometric analysis revealed a 10% decrease in the ALV-A-GFP infection rate in DF-1-MARCH2 cells compared to that in wild-type (WT) cells (Fig. 1G). To substantiate the regulatory effect of MARCH2 on the WT ALV-A strain, DF-1-ctl and DF-1-MARCH2 cells were infected with ALV-A (RAV-1 strain). Western blotting demonstrated a significant reduction in ALV-A protein (p27 and gp85) levels in DF-1-MARCH2 cells compared to that in DF-1-ctl cells (Fig. 1H through J). RT-qPCR showed a 1.32- and 4.29-fold decrease in the levels of mRNA encoded by the ALV-A viral genome in *MARCH2*-overexpressing cells at 48 and 120 h post-infection, respectively (Fig. 1K). Additionally, infectivity of the released virus was assessed using the tissue culture infectious dose (TCID_50_) method. Here, the overexpression of MARCH2 resulted in a reduction in viral titers from 3.67 log10 to 3.13 log10 at 48 h post-infection and from 5.76 log10 to 4.53 log10 at 120 h post-infection (Fig. 1L). Taken together, these findings indicate that *MARCH2* overexpression significantly inhibits ALV-A replication.

**Fig 2:**
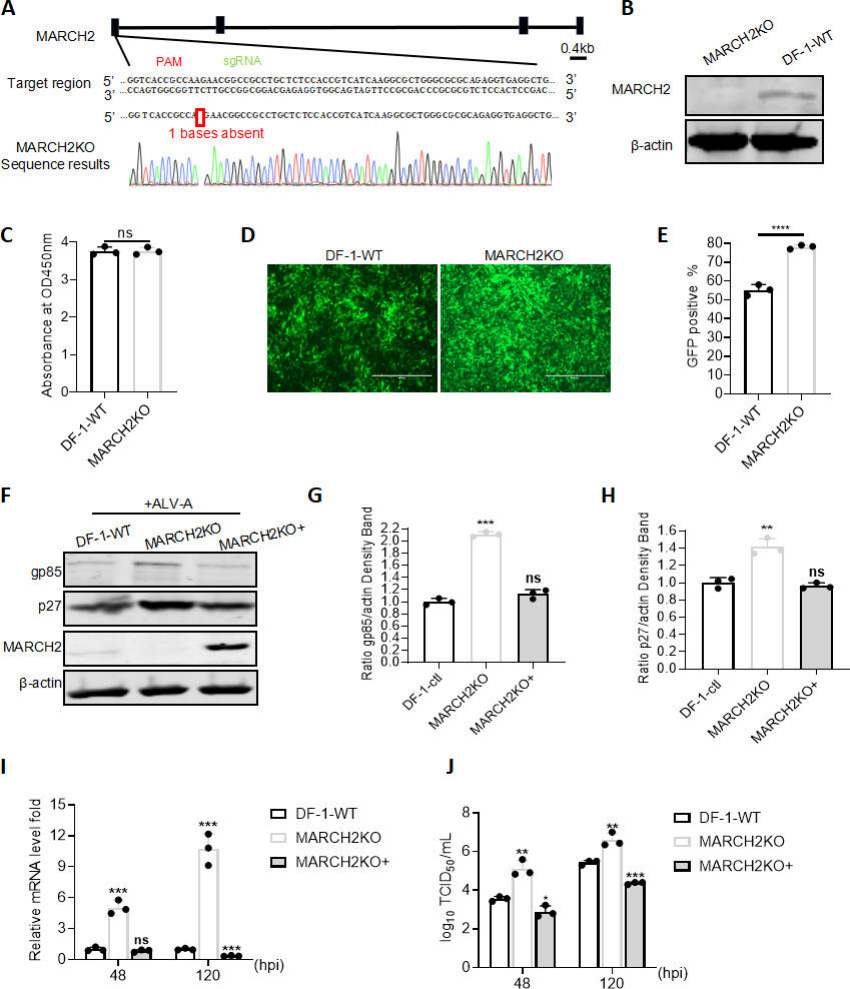
MARCH2 knockout (MARCH2KO) enhances ALV-A replication. (**A**) Schematic diagram of the construction of MARCH2KO DF-1 cells and sequence analysis of the MARCH2KO cell lines. (**B**) Validation of MARCH2KO cell lines by western blotting with an anti-MARCH2 antibody. (**C**) Comparative analysis of cell viability between wild-type (WT) and MARCH2KO DF-1 cell lines. (**D and E**) Effect of MARCH2 deficiency on ALV-A-GFP replication. (**D**) Evaluation of viral infectivity at 120 h post-infection with ALV-A-GFP, visualized under a fluorescence microscope. Scale bar = 125 µm. (**E**) Quantification of viral infectivity via flow cytometry, assessing the percentage of GFP-positive cells following ALV-A-GFP infection for 120 h. (**F–J**) Consequences of MARCH2KO for WT ALV-A replication. (**F**) p27 and gp85 protein expression levels were determined via western blotting post-ALV-A infection for 120 h. (**G**) Relative intensities of gp85 were normalized with β-actin in the cell lysis. (**H**) Relative intensities of p27 were normalized with β-actin in the cell lysis. (**I**) Calculation of the relative fold change in ALV-A genomic mRNA levels in cell lysates using RT-qPCR post-ALV-A infection for 48 or 120 h. (**J**) Assessment and graphical representation of the released viral titer based on the tissue culture infectious dose (TCID_50_) assay post-ALV-A infection for 48 or 120 h. *P < 0.05, **P < 0.01, ***P < 0.001, and ****P < 0.0001; ns, no significant difference.

To further determine the influence of *MARCH2* on ALV-A replication, a DF-1 cell line with MARCH2 knockout (MARCH2KO) was established using CRISPR/Cas9 technology. Sequence analysis revealed a nucleotide deletion in the endogenous *MARCH2* ORF, indicating the successful generation of this cell line (Fig. 2A). Western blotting analysis further confirmed the successful knockout of MARCH2 at the protein level using an anti-MARCH2 antibody (Fig. 2B). Additionally, the Cell Counting Kit 8 (CCK-8) assay results indicated that MARCH2KO did not affect cell viability (Fig. 2C). To assess the influence of the MARCH2KO on ALV-A replication, WT DF-1 and MARCH2KO cells were infected with ALV-A-GFP. Fluorescence observations revealed that MARCH2KO led to increased ALV-A-GFP infectivity (Fig. 2D), and a flow cytometric analysis demonstrated a 23% increase in the ALV-A-GFP infection rate in MARCH2KO cells compared to that in WT cells (Fig. 2E). To further validate the regulatory role of MARCH2 in the WT ALV-A strain and eliminate potential off-target effects associated with MARCH2KO, we re-expressed *MARCH2* in MARCH2KO cell lines (designated as MARCH2KO+). Subsequently, WT DF-1 cells, MARCH2KO cells, and MARCH2KO+ cells were all infected with the ALV-A RAV-1 strain. Western blotting results showed a significant increase in ALV-A p27 and gp85 protein levels in MARCH2KO cells compared to those in WT and MARCH2KO+ cells (Fig. 2F through H). Consistent with these protein levels, RT-qPCR results indicated a 5.04- and 10.70-fold increase in the levels of mRNA encoded by the ALV-A viral genome in MARCH2KO cells compared to those in WT cells at 48 and 120 h post-infection, respectively, and this promoting effect was abolished when *MARCH2* was re-expressed in MARCH2KO cells (Fig. 2I). Additionally, the viral titer in the cell supernatant was measured using the TCID_50_ method. Here, the knockout of MARCH2 led to an increase in viral titers from 3.57 log10 to 5.11 log10 at 48 h post-infection and from 5.53 log10 to 6.59 log10 at 120 h post-infection (Fig. 2J). Conversely, when *MARCH2* was re-expressed in MARCH2KO cells, the viral titer significantly decreased. Taken together, these results suggest that MARCH2KO significantly enhances ALV-A replication, highlighting the importance of MARCH2 as a negative regulator of ALV infection.

### MARCH2 impairs ALV replication by selectively targeting viral protein stability

**Fig 3:**
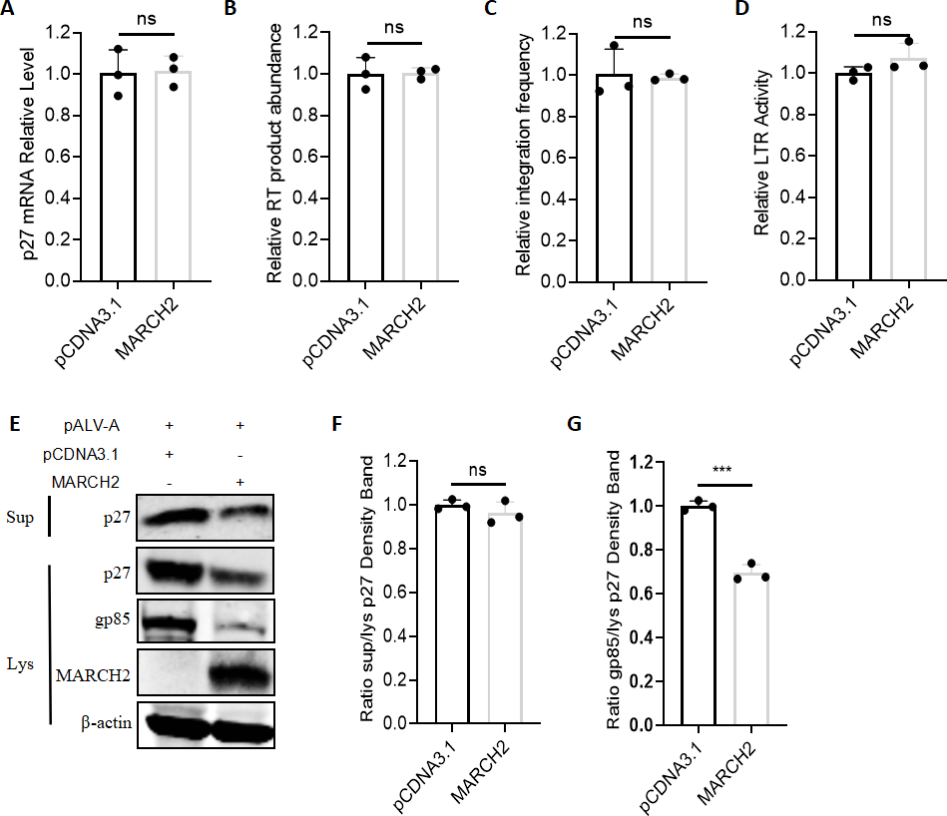
MARCH2 impairs ALV replication by selectively targeting viral protein stability. (**A**) After infecting ALV-A on ice for 2 h and 37°C for 24 h, RT-qPCR was conducted to quantify the level of p27 mRNA in DF-1 cells as an indicator of viral entry. The relative fold change in p27 mRNA expression was normalized to that in cells not transfected with the MARCH2 expression plasmid. (**B**) After 8 h of ALV-A infection, RT-qPCR was conducted to quantify the level of p27 mRNA in DF-1 cells as an indicator of reverse transcription. The relative fold change in p27 mRNA expression was normalized to that in cells not transfected with the MARCH2 expression plasmid. (**C**) After 24 h of ALV-A infection, RT-qPCR was conducted to quantify the level of p27 mRNA of DF-1 genomes as an indicator of integration products. The relative fold change in p27 mRNA expression was normalized to that in cells not transfected with the MARCH2 expression plasmid. (**D**) Evaluation of the effect of MARCH2 on LTR-driven reporter gene expression using a luciferase assay. (**E**) Virions in the supernatant and cell lysates of DF-1 cells transfected with the ALV-A infectious clone were analyzed by western blotting. (**F**) Relative intensities of p27 in the supernatant were normalized with p27 in the cell lysis. (**G**) Relative intensities of gp85 were normalized with p27 in the cell lysis. ***P < 0.001; ns, no significant difference.

To determine how MARCH2 affects ALV replication, we examined its effect on different stages of the ALV replication cycle. DF-1 cells were transfected with MARCH2 and subsequently infected with ALV-A. Viral products corresponding to various replication stages were analyzed, and the results indicated that MARCH2 expression did not affect viral entry (Fig. 3A), reverse transcription (Fig. 3B), or integration (Fig. 3C). Furthermore, to assess whether MARCH2 influences ALV promoter activity, DF-1 cells were co-transfected with MARCH2 and a luciferase reporter plasmid under the control of the ALV-A promoter. Luciferase assays revealed that MARCH2 did not alter ALV-A promoter activity (Fig. 3D). To evaluate whether MARCH2 affects viral protein expression and virion assembly/release, DF-1 cells were co-transfected with MARCH2 and an ALV-A infectious clone. At 48 h post-transfection, both cell lysates and culture supernatants were collected. Levels of intracellular viral proteins and extracellular virion release were quantified. The results showed that although MARCH2 caused an overall reduction in viral protein levels, it did not alter the ratio of extracellular to intracellular viral structural protein p27, indicating that MARCH2 does not inhibit viral particle release (Fig. 3E and F). Notably, the intracellular level of gp85 decreased more markedly than that of p27 (Fig. 3E and G), suggesting that MARCH2 may impair ALV replication by selectively targeting viral protein stability.

### MARCH2 interacts with gp85

**Fig 4:**
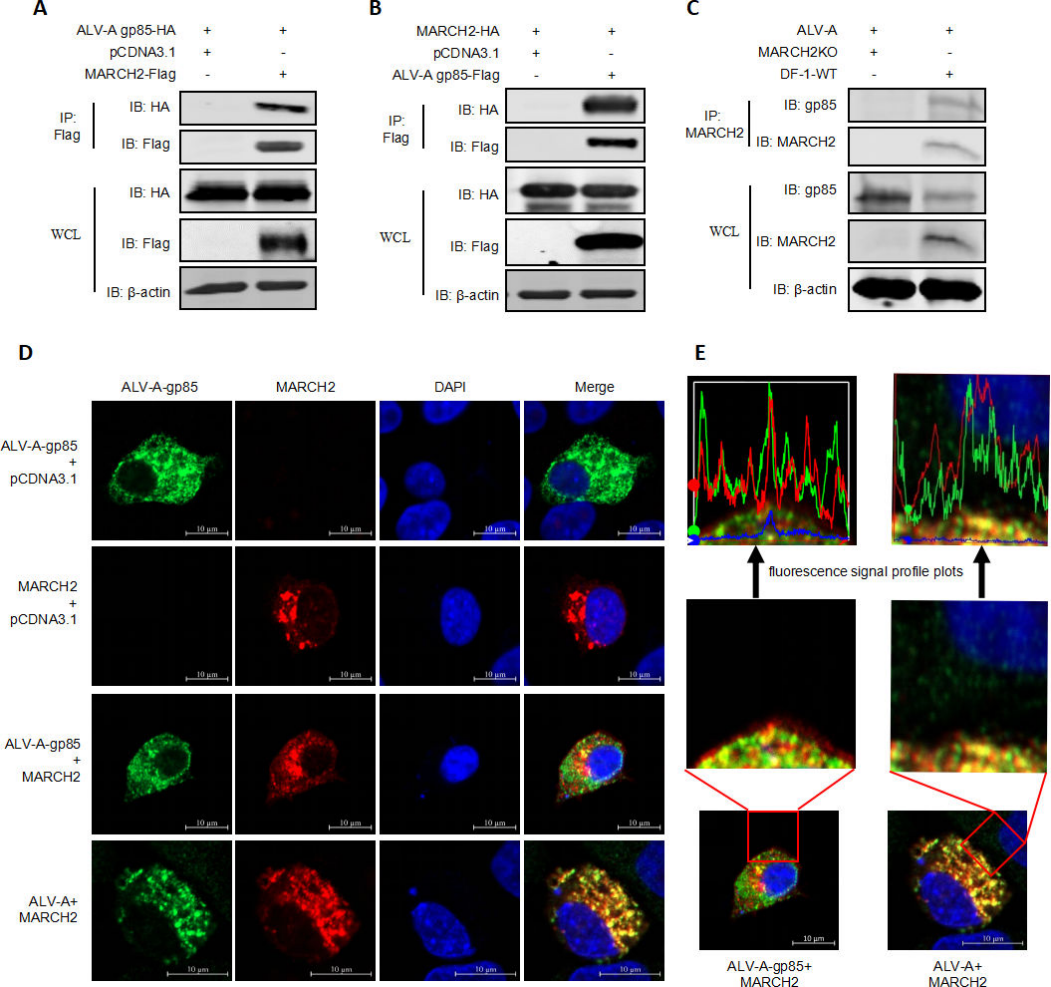
MARCH2 interacts with gp85. (**A and B**) Assessment of the interaction between MARCH2 and the gp85 via western blotting. (**C**) The interaction between MARCH2 and gp85 was examined in ALV-infected DF-1 cells using co-IP with an anti-MARCH2 antibody. (**D**) Utilization of confocal microscopy to demonstrate the co-localization of MARCH2 (red) with gp85 (green). (**E**) The co-localization of MARCH2 and gp85 was visualized and magnified using fluorescence signal profile plots. Scale bar = 10 µm.

The MARCH family proteins are a type of E3 Ub ligase that can regulate the ubiquitination of membrane proteins. Numerous studies have demonstrated that MARCH family proteins interact with the envelope proteins of enveloped viruses (32, 33). For example, MARCH2 directly targets the envelope proteins of enveloped viruses such as human immunodeficiency virus, Ebola virus, Nipah virus, Chikungunya virus, and lymphocytic choriomeningitis virus for degradation (26, 29, 34, 35). To ascertain whether MARCH2 suppresses ALV-A replication through a direct interaction with the ALV-A surface glycoprotein (gp85), co-immunoprecipitation (co-IP) experiments were conducted. The results revealed a strong interaction between MARCH2 and gp85 (Fig. 4A), and reverse co-IP results corroborated this interaction (Fig. 4B). Importantly, during ALV-A virus infection, MARCH2 was found to interact strongly with the viral gp85 protein (Fig. 4C). Pull-down assay result showed that MARCH2 interacts strongly with the viral gp85 protein (Fig. S2). Furthermore, confocal microscopy confirmed the co-localization of MARCH2 and gp85 in the cytoplasm, with a similar pattern observed for MARCH2 and viral gp85 during ALV-A infection (Fig. 4D), characterized by similar fluorescence response signal patterns (Fig. 4E). Collectively, these results indicate that MARCH2 interacts with gp85, suggesting a direct molecular mechanism by which MARCH2 regulates ALV-A replication.

### MARCH2 promotes gp85 degradation via the proteosome

**Fig 5:**
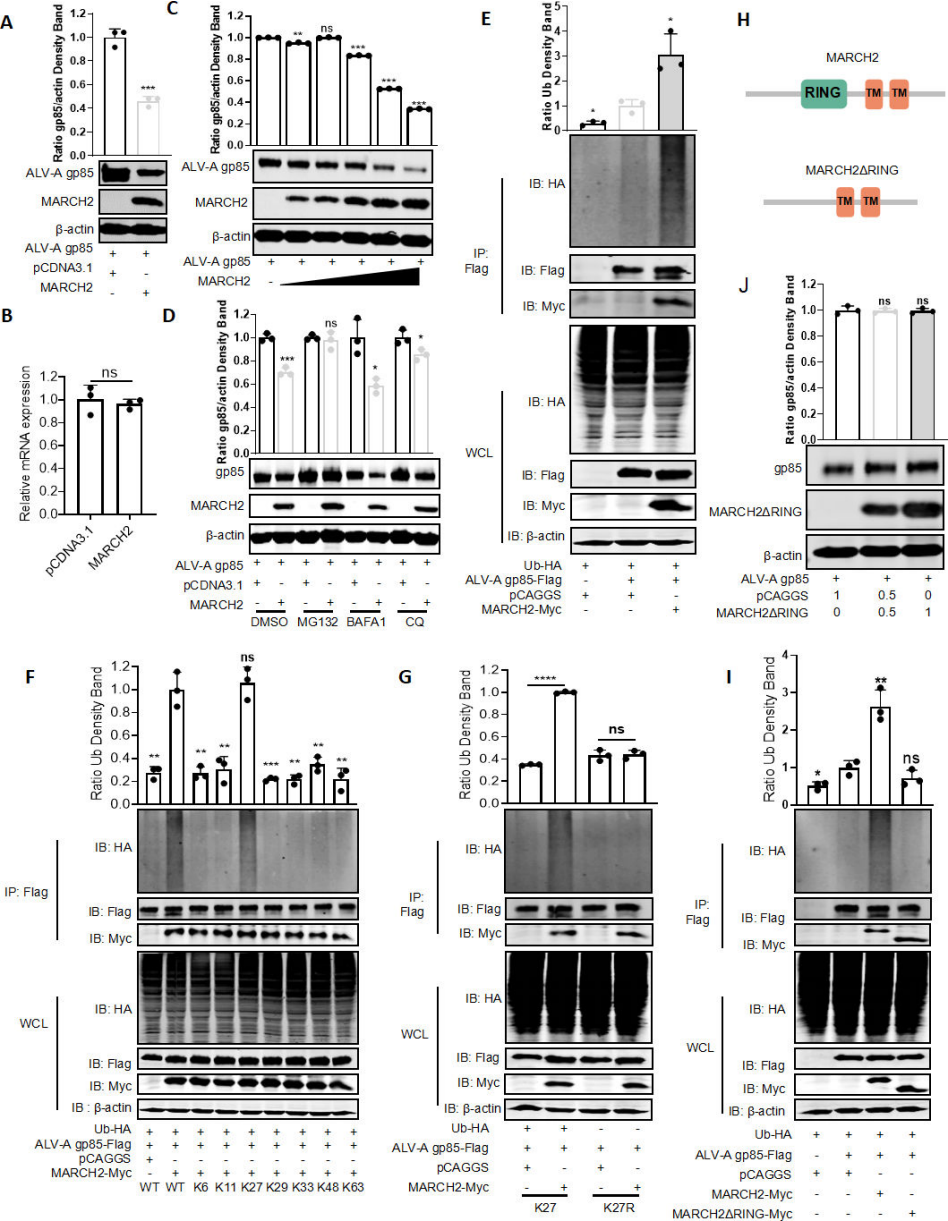
MARCH2 promotes gp85 degradation via the proteasomal pathway. (**A and B**) MARCH2 negatively regulates the expression levels of gp85. (**A**) Expression levels of gp85 were quantified through western blotting. Relative intensities of gp85 were normalized with β-actin in the cell lysis. (**B**) Levels of *gp85* mRNA were assessed using RT-qPCR. (**C**) Expression levels of gp85 were assessed following co-transfection with varying concentrations of MARCH2 expression plasmids using western blotting. Relative intensities of gp85 were normalized with β-actin in the cell lysis. (**D**) Expression levels of gp85 were evaluated following co-transfection with MARCH2 expression plasmids and subsequent treatment with dimethyl sulfoxide (DMSO) as a negative control, MG132, bafilomycin A1 (BAFA1), or chloroquine phosphate (CQ) using western blotting. Relative intensities of gp85 were normalized with β-actin in the cell lysis. (**E**) The ubiquitination of gp85 induced by MARCH2 was detected via western blotting. Relative intensities of Ub were normalized with WT Ub co-transfected with ALV-A gp85. (**F**) Determination of the type of gp85 ubiquitination catalyzed by MARCH2. gp85 plasmids and different Ub plasmids were co-transfected with MARCH2 plasmids. Following this, the lysates were analyzed via western blotting. Relative intensities of Ub were normalized with WT Ub co-transfected with MARCH2. (**G**) Determine MARCH2 catalyzes K27-linked ubiquitination of gp85. Cells were co-transfected with plasmids encoding gp85, MARCH2, and either wild-type (WT) Ub or the K27R Ub mutant. Cell lysates were then analyzed by western blotting to detect gp85 ubiquitination. Relative intensities of Ub were normalized with Ub co-transfected with pCAGGS. (**H–J**) The degradation of gp85 induced by MARCH2 depended on its E3 ubiquitin ligase activity. (**H**) Schematic representation of the structure of MARCH2 and MARCH2ΔRING. (**I**) The effect of the MARCH2ΔRING mutation on the ubiquitination of gp85 was determined using western blotting. Relative intensities of Ub were normalized with WT Ub co-transfected with ALV-A gp85. (**J**) The effect of the MARCH2ΔRING mutation on the expression levels of gp85 was determined using western blotting. Relative intensities of gp85 were normalized with β-actin in the cell lysis. *P < 0.05, **P < 0.01, and ***P < 0.001; ns, no significant difference.

To further elucidate the role of the MARCH2-gp85 interaction in the MARCH2-mediated regulation of ALV-A replication, we initially explored the effect of MARCH2 on gp85 expression levels by co-transfecting a MARCH2 plasmid and gp85 plasmid into DF-1 cells. Western blotting revealed that MARCH2 suppressed gp85 expression (Fig. 5A). To elucidate the mechanism by which MARCH2 inhibits gp85 protein expression, we co-transfected DF-1 cells with varying amounts of MARCH2 expression plasmids along with gp85 expression plasmids and subsequently assessed the transcriptional and translational levels of gp85. RT-qPCR results indicated no change in *gp85* mRNA levels, regardless of MARCH2 co-expression (Fig. 5B), whereas significant gp85 protein degradation occurred in a dose-dependent manner as the expression of MARCH2 was increased (Fig. 5C). To ascertain the pathway through which MARCH2 induces the degradation of gp85, DF-1 cells were co-transfected with MARCH2 and gp85 for 24 h, followed by treatment with a proteasome inhibitor (MG132) and lysosome inhibitors (bafilomycin A1 and chloroquine phosphate) for 12 h. In contrast to observations based on the dimethyl sulfoxide control group, after treatment with the proteasome inhibitor MG132, MARCH2 could not effectively reduce the expression levels of gp85, whereas treatment with a lysosome inhibitor still resulted in MARCH2-induced gp85 degradation (Fig. 5D). These results suggest that MARCH2 degrades gp85 in a dose- and proteasome-dependent manner.

The post-translational modifications of proteins by Ub and their subsequent degradation via the Ub–proteasome system comprise a crucial regulatory mechanism used for maintaining cellular protein homeostasis (36, 37). To elucidate the ubiquitination status of gp85, pFlag-gp85, pMyc-MARCH2, and pHA-Ub plasmids were co-transfected into HEK293T cells. Western blotting showed that MARCH2 significantly increased the ubiquitination of gp85, as determined via IP and ubiquitination assays (Fig. 5E). To further confirm the type of the polyubiquitin chain bound to gp85, catalyzed by MARCH2, we constructed a pHA-Ub WT plasmid and eight mutant Ub plasmids (K6, K11, K27, K29, K33, K48, and K63) and co-transfected them with pMyc-MARCH2 and pFlag-gp85 plasmids. IP and ubiquitination assay results indicated that the ubiquitination levels of gp85 were enhanced by MARCH2 in the presence of K27 Ub (Fig. 5F). In contrast, the ubiquitination of gp85 was impaired when co-transfected with pHA-Ub (K27R) (Fig. 5G), suggesting that MARCH2 mediates the K27-linked polyubiquitination of gp85.

MARCH proteins are characterized by a conserved RING domain that is recognized for its E3 Ub ligase function in the ubiquitination cascade. To determine whether the ubiquitination of gp85 is dependent on the ubiquitinase activity of MARCH2, a MARCH2ΔRING mutant, devoid of the RING domain, was engineered (Fig. 5H). IP and ubiquitination assays revealed a significant reduction in gp85 ubiquitination in the presence of this mutant, indicating that the RING domain is indispensable for MARCH2 Ub ligase activity and gp85 ubiquitination (Fig. 5I). Subsequent studies were conducted to ascertain the effect of the MARCH2 RING-domain-deletion mutant on the degradation of gp85. Western blotting analysis indicated that gp85 expression levels remained unaltered after co-transfection with varying quantities of the MARCH2ΔRING plasmids (Fig. 5J). Collectively, these findings indicate a critical role for MARCH2 Ub ligase activity in the proteolytic regulation of gp85 and its antiviral effect on ALV.

### K282 of gp85 is a ubiquitination target site for MARCH2

**Fig 6:**
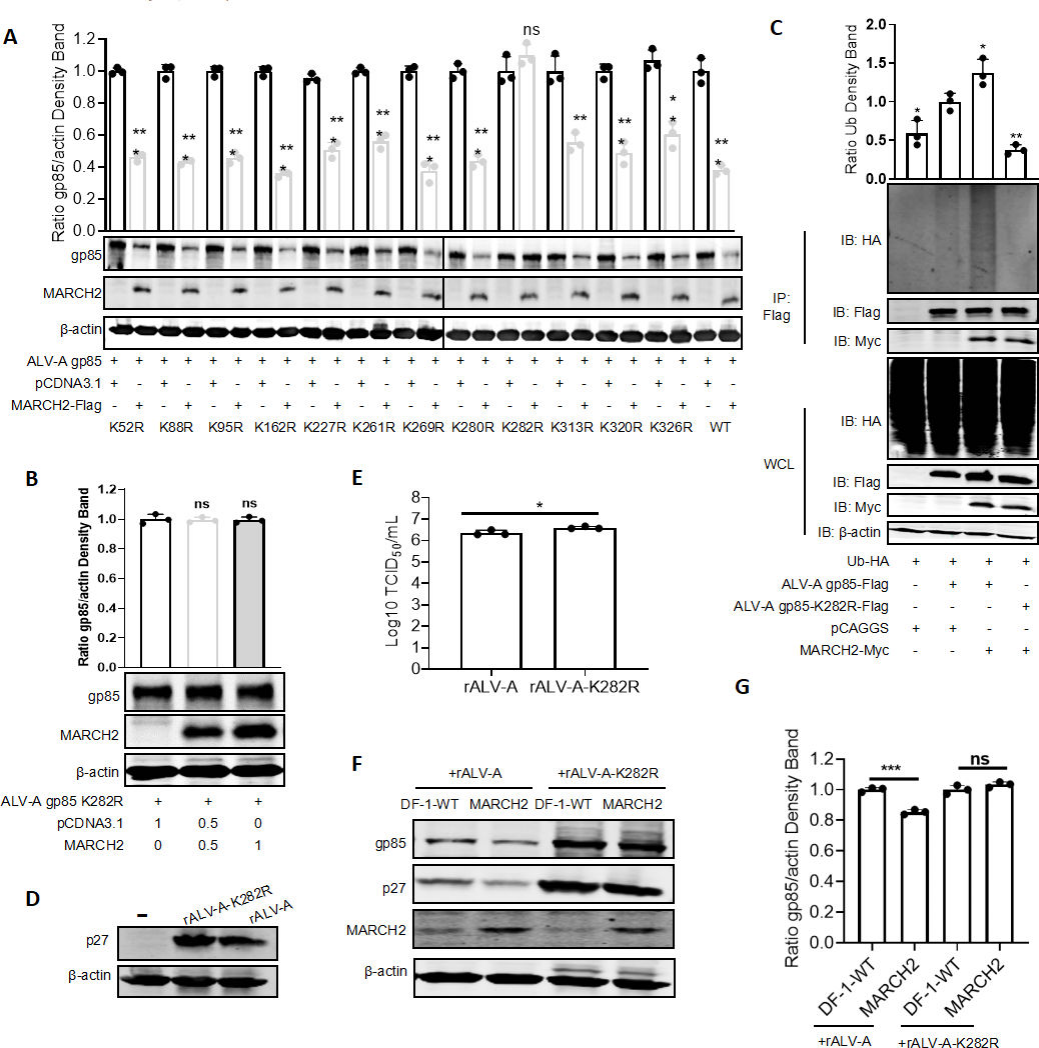
K282 of gp85 is a ubiquitination site targeted by MARCH2. (**A**) Distinct gp85 plasmids, including both wild-type (WT) and mutant forms, were co-transfected with MARCH2 plasmids. Following this, the lysates were analyzed via western blotting. Relative intensities of gp85 were normalized with β-actin in the cell lysis.(**B**) The expression levels of the K282R mutant form of gp85, co-transfected with varying concentrations of MARCH2 expression plasmids, were quantified using western blotting. Relative intensities of gp85 were normalized with β-actin in the cell lysis. (**C**) Western blotting was performed to assess the ubiquitination levels of gp85 K282R. Relative intensities of Ub were normalized with WT Ub co-transfected with ALV-A gp85. (**D**) Viral rescue was confirmed by detecting the expression of the viral p27 protein using western blotting. (**E**) Viral rescue was validated through the quantification of viral titers using the tissue culture infectious dose (TCID_50_) assay. (**F**) Effect of MARCH2 overexpression on the replication of ALV-A and its K282R mutant form (rALV-A-K282R) through western blotting analysis of viral p27 and gp85 protein expression post-infection for 120 h. (**G**) Relative intensities of gp85 were normalized with β-actin. *P < 0.05, **P < 0.01, and ***P < 0.001; ns, no significant difference.

Ubiquitylation involves the attachment of Ub to lysine residues on target proteins and is a critical step in protein degradation. To accurately identify the lysine residue in gp85 targeted by MARCH2 for proteasomal degradation, we constructed corresponding arginine-mutant expression plasmids based on the 12 lysine residues of gp85, namely pFLAG-gp85K52R, pFLAG-gp85K88R, pFLAG-gp85K95R, pFLAG-gp85K162R, pFLAG-gp85K227R, pFLAG-gp85K261R, pFLAG-gp85K269R, pFLAG-gp85K280R, pFLAG-gp85K282R, pFLAG-gp85K313R, pFLAG-gp85K320R, and pFLAG-gp85K326R. These mutants were co-transfected with MARCH2 and subjected to western blotting analysis, and the mutant with a substitution at the ninth lysine residue (K282R) of gp85 was resistant to MARCH2-mediated degradation (Fig. 6A and B). Additionally, IP and ubiquitination assays revealed that the K282R mutation in gp85 rendered it incapable of being ubiquitinated by MARCH2 (Fig. 6C). To further determine whether the K282R mutation in gp85 affects ALV-A replication, we first rescued the WT ALV-A (rALV-A) and a mutant ALV-A lacking the critical ubiquitination site at K282 (rALV-A-K282R) in DF-1 cells. Western blotting revealed strong p27 expression after rALV-A and rALV-A-K282R infection in DF-1 cells, indicating that the two viruses were rescued successfully (Fig. 6D). The virus titer assays also confirmed the successful rescue of both viruses (Fig. 6E). Additionally, the results of the virus infection experiments indicated that MARCH2 did not induce degradation of the gp85 protein in the mutant virus and had no inhibitory effect on viral replication at 120 h post-infection (Fig. 6F and G). Taken together, these findings indicate that disruption of the K282 ubiquitination site of gp85 increases the replication efficiency of ALV-A.

### MARCH2 inhibits the replication of multiple subgroups of ALVs

**Fig 7:**
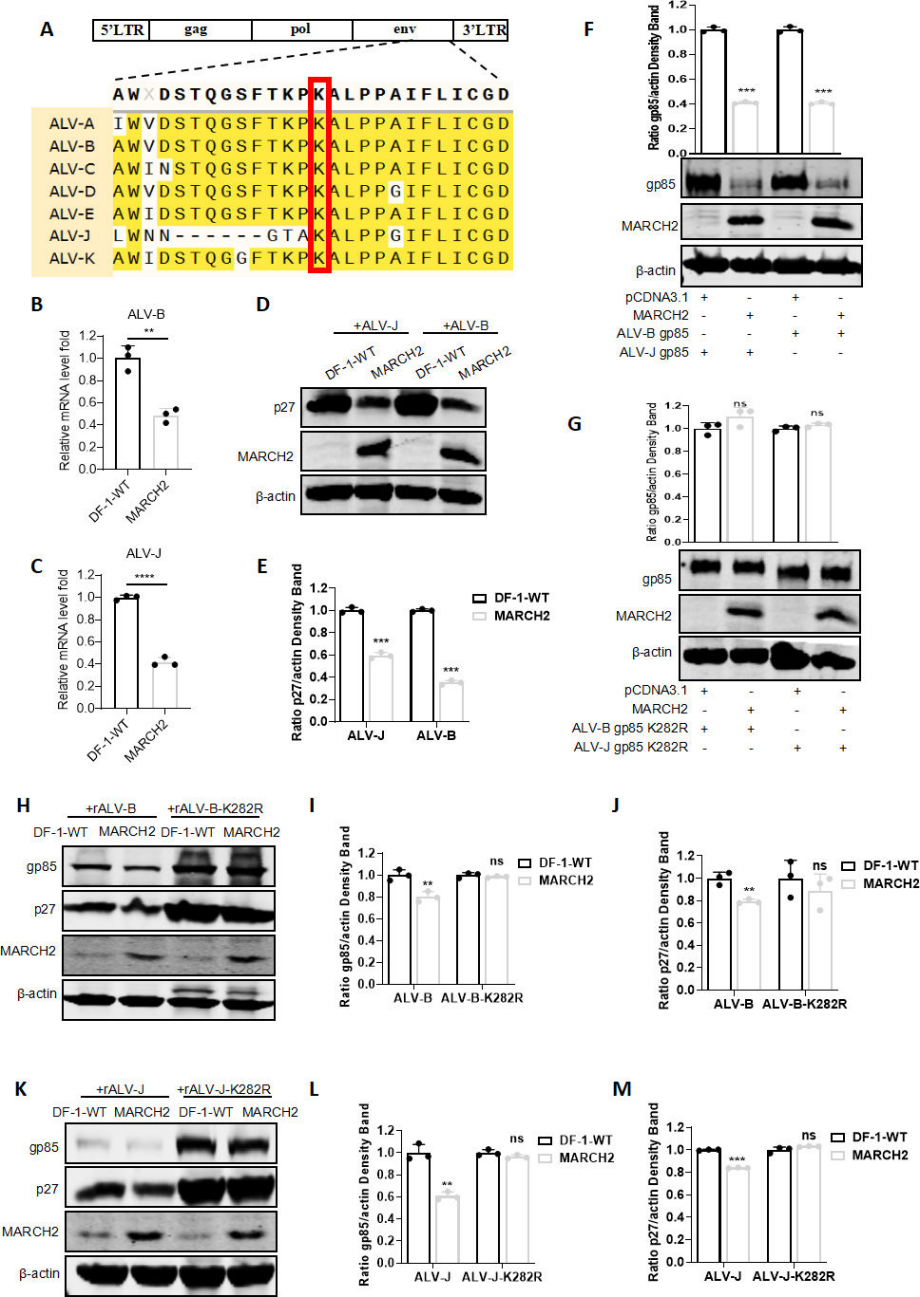
MARCH2 inhibits the replication of multiple subgroups of ALVs. (**A**) Amino acid sequence comparison of gp85 proteins from ALV-A, ALV-B, ALV-C, ALV-D, ALV-E, ALV-J, and ALV-K. (**B–E**) Effect of MARCH2 overexpression on the replication of ALV-B and ALV-J. (**B and C**) The relative fold changes in the levels of mRNA encoded by the ALV-B (**B**) and ALV-J (**C**) genomes in cellular samples were determined via RT-qPCR. (**D**) Expression levels of the p27 protein were ascertained through western blotting post-infection for 120 h. (**E**) Relative intensities of p27 were normalized with β-actin in the cell lysis. (**F**) Expression levels of the gp85 protein from both ALV-B and ALV-J were assessed via western blotting after co-transfection with gp85 and MARCH2 expression plasmids. Relative intensities of gp85 were normalized with β-actin in the cell lysis. (**G**) Expression levels of the K282R mutant form of gp85 from ALV-B and ALV-J were determined via western blotting after co-transfection with gp85 and MARCH2 expression plasmids. Relative intensities of gp85 were normalized with β-actin in the cell lysis. (**H**) Effect of MARCH2 overexpression on the replication of ALV-B and its K282R mutant form (rALV-B-K282R) through western blotting analysis of viral p27 and gp85 protein expression post-infection for 120 h. (**I**) Relative intensities of gp85 were normalized with β-actin in the cell lysis. (**J**) Relative intensities of gp85 were normalized with β-actin in the cell lysis. (**K**) Effect of MARCH2 overexpression on the replication of ALV-J and its K282R mutant form (rALV-J-K282R) through western blotting analysis of viral p27 and gp85 protein expression post-infection for 120 h. (**L**) Relative intensities of gp85 were normalized with β-actin in the cell lysis. (**M**) Relative intensities of gp85 were normalized with β-actin in the cell lysis. **P < 0.01, ***P < 0.001, and ****P < 0.0001; ns, no significant difference.

Despite the diversity of the ALV subgroups and the relatively low amino acid homology of gp85 across these subgroups (38), the lysine residue at position 282 (K282) is conserved among the various poultry-infecting ALV subgroups (Fig. 7A). This suggests that MARCH2 may regulate the replication of different ALV subgroups. RT-qPCR showed a decrease in the levels of mRNA encoded by the ALV-B (Fig. 7B) and ALV-J (Fig. 7C) viral genomes in MARCH2-overexpressing cells. In agreement with this, Western blotting results demonstrated a reduction in ALV-B and ALV-J protein (p27) levels in DF-1-MARCH2 cells relative to those in WT DF-1 cells (Fig. 7D and E). To validate the regulatory effect of MARCH2 on gp85 across different ALV subgroups, we co-transfected MARCH2 with expression plasmids encoding gp85 from either ALV-B or ALV-J into DF-1 cells, followed by western blotting analysis. The results demonstrated that MARCH2 significantly reduced the protein levels of gp85 from both ALV-B and ALV-J (Fig. 7F). To determine whether the degradation of gp85 by MARCH2 is dependent on the K282 site, we constructed expression plasmids carrying the K282R point mutation in the gp85 gene for both ALV-B and ALV-J. Subsequent co-transfection with MARCH2 and western blotting analysis demonstrated that the degradation of gp85 was completely abolished in both K282R mutants (Fig. 7G). Additionally, WT ALV-B and ALV-J (rALV-B and rALV-J), as well as mutant ALV-B and ALV-J lacking the critical ubiquitination site at K282 (rALV-B-K282R and rALV-J-K282R), were rescued. Virus infection experiments indicated that, at 120 h post-infection, MARCH2 neither degraded gp85 nor suppressed viral replication in the K282R mutant ALV-B (Fig. 7H through J) and ALV-J (Fig. 7K through M). These findings indicate that MARCH2 modulates the replication of different ALV subgroups by targeting the ubiquitination of K282 on gp85, facilitating the subsequent degradation of gp85, and thereby inhibiting ALV replication.

## DISCUSSION

**Fig 8:**
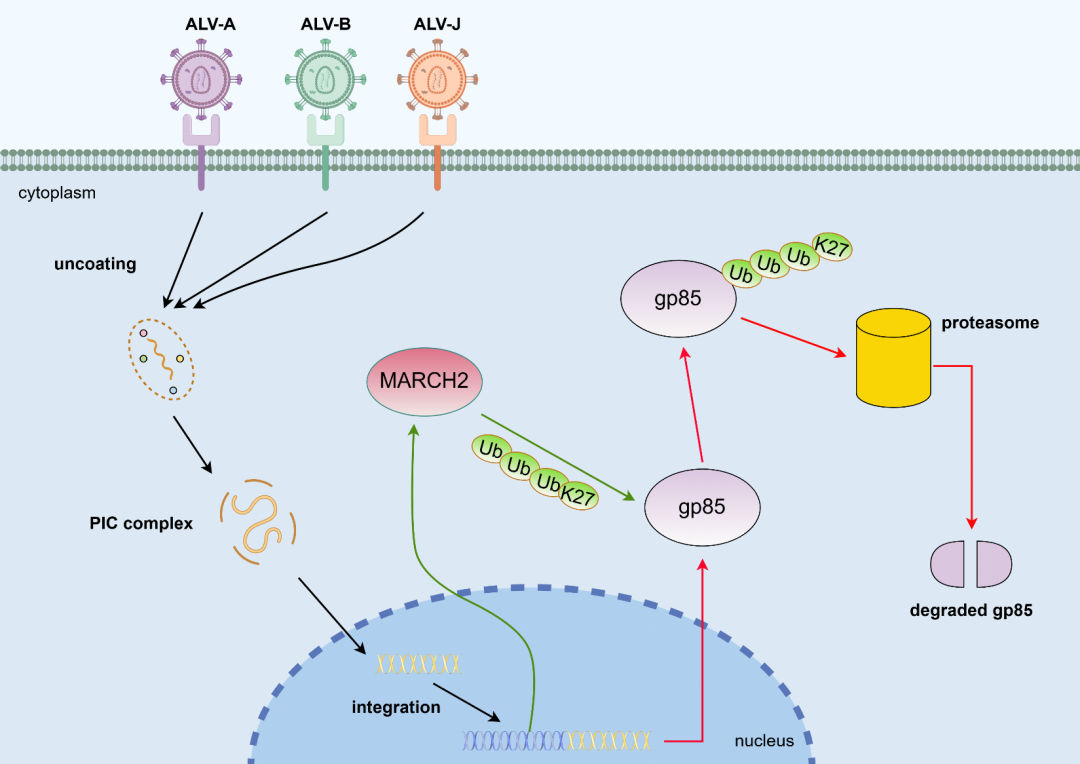
Model by which MARCH2 suppresses ALV replication by targeting gp85 for degradation. MARCH2 was found to inhibit ALV replication by directly interacting with the viral protein gp85, promoting its ubiquitination and subsequent degradation mediated by the proteasome.

Owing to the inefficiency of eradication strategies and the lack of effective vaccines, ALV remains a significant disease that seriously endangers the safety of breeding sources. The upregulation of the expression of many host antiviral factors induced by ALV infection further results in resistance to viral infection, and these host factors are important targets for formulating new ALV prevention and control strategies. Our research identified MARCH2 as a host restriction factor for various ALV subgroups and demonstrated that it inhibits the replication of different ALV subgroups by directly interacting with the gp85 protein, promoting K27-linked ubiquitination at the K282 site and its subsequent degradation via the proteasome pathway (Fig. 8). This discovery provides a potential target for the development of strategies to control different ALV subgroups.

The MARCH family of proteins has been extensively implicated in the replication of various viruses and plays a dual role in modulating their life cycles (21–27). For example, MARCH8 interacts with the NS2 protein of hepatitis C virus, facilitating its ubiquitination and promoting viral budding (31). Conversely, MARCH8 also engages with the M2 protein of the influenza A virus, leading to its degradation, thereby inhibiting viral replication (39). In this study, we revealed an intriguing aspect of MARCH2 protein expression during ALV-A infection. Specifically, the significant upregulation of MARCH2 expression was observed post-infection, suggesting that MARCH2 may function as a sentinel protein activated in response to ALV-A infection. This upregulation could potentially suppress ALV-A replication by controlling the availability of gp85, a critical component required for ALV-A virus assembly. Thus, MARCH2 may play a more pivotal role in ALV-A infection-related processes than the other MARCH proteins. This is the first study to show that MARCH2 is involved in regulating avian retroviral replication.

The MARCH2 protein, which functions as an E3 Ub ligase, plays a pivotal role in controlling retroviral replication (40–42). Specifically, MARCH2 downregulates the expression of HIV-1 Env glycoproteins on the cell surface, thereby preventing their integration into newly formed virions (34, 35). This reduction in Env protein levels leads to a decrease in viral entry efficiency, which, in turn, reduces the ability of the virus to infect host cells. In contrast, during murine leukemia virus infection, MARCH1 and MARCH8, rather than MARCH2, were recognized as key players in reducing viral infection that function by directing the degradation of viral envelope glycoproteins (43). Our research further revealed that chicken MARCH2 directly interacts with the gp85 protein of ALV-A, enhancing its ubiquitination and leading to its degradation, mediated by the proteasome, thereby curbing ALV-A replication. These findings underscore the intricate and nuanced antiviral mechanisms employed by MARCH proteins, in which unique members of the MARCH family are used to counteract viral replication via specific inhibitory mechanisms, thereby highlighting the versatility and complexity of these host defense proteins.

The most common ALV subgroups affecting poultry are ALV-A, ALV-B, and ALV-J. The primary distinction among these subgroups is gp85, which plays a crucial role in viral invasion. Our research identified gp85 as a critical target for MARCH2 during the inhibition of ALV-A replication. Specifically, MARCH2 was found to promote the ubiquitination and subsequent degradation of gp85, with lysine 282 (K282) being the principal site for this modification. Notably, the K282 site is conserved across various ALV subgroups, and mutations at this site can block the MARCH2-mediated degradation of gp85. Our viral infection experiments further indicated that MARCH2 significantly inhibits ALV subgroups A, B, and J, as these subgroups are the most common and pathogenic in poultry. These insights not only underscore the therapeutic potential of targeting the K282 site of the ALV gp85 protein but also highlight the necessity of future epidemiological surveys to place a greater emphasis on amino acid mutations at the ALV gp85 K282 site. Focus on this could provide an early warning system for outbreaks.

Host organisms primarily suppress viral replication through the degradation of viral proteins through the lysosomal or proteasomal pathways (44–46). Several MARCH family E3 Ub ligases, such as MARCH8 and MARCH1, are known to mediate viral protein degradation through the lysosomal pathway. MARCH8 targets IAV M2 (39) and PEDV N proteins (47), and MARCH1 targets the MLV envelope glycoprotein (43). In contrast, our study shows that MARCH2 specifically promotes the degradation of ALV-A gp85 via the proteasomal pathway. This process is strictly dependent on the MARCH2-induced ubiquitination of gp85. The preferential use of the proteasomal rather than the lysosomal pathway for gp85 degradation may be attributed to distinct substrate characteristics, such as the subcellular localization, structural features, or affinity for proteasome-associated receptors, which require further experimental validation. These findings not only underscore the mechanistic diversity within the MARCH protein family in targeting viral substrates but also reveal a novel proteasomal function of MARCH2 in restricting retroviral replication.

In conclusion, the present results corroborate the important role of MARCH2 in combating ALV infections. Usually, MARCH2 inhibits viral replication by suppressing the membrane localization of viral glycoproteins in enveloped viruses. However, our results indicated that MARCH2 directly targets the envelope glycoprotein of ALV for degradation, thereby exerting its antiviral effects. These findings not only suggest a novel mechanism by which host factors, such as the MARCH family of proteins, regulate the replication of enveloped viruses but also offer new insights into the prevention and control of ALV.

## MATERIALS AND METHODS

### Cells, viruses, and plasmids

The HEK 293T and DF-1 cell lines were obtained from the American Type Culture Collection and cultured in Dulbecco’s modified Eagle medium (DMEM; Basal Media, L110KJ) supplemented with 10% fetal bovine serum (FBS; Sigma-Aldrich, F0193). The DF-1 cells were incubated in a humidified environment with 5% CO_2_ at a temperature of 38.5°C. In contrast, HEK293T cells were maintained under the same CO_2_ conditions at a temperature of 37°C. The ALV-A prototype strain RAV-1 was kindly provided by Venugopal Nair (Pirbright Institute, Pirbright, UK) and propagated in DF-1 cells as previously described (48, 49).

The *MARCH* gene was PCR-amplified from cDNA derived from DF-1 cells and subsequently cloned into the pCAGGS vector with a C-terminal Flag, HA, or Myc epitope tag. Similarly, the *gp85* gene was amplified from the genomic DNA of ALV-A, ALV-B, and ALV-J strains and cloned into pCDNA3.1, along with the addition of a C-terminal Flag, HA, or Myc tag. Truncated or point-mutated expression plasmids were constructed based on the aforementioned vectors using fusion PCR. All recombinant constructs were confirmed via DNA sequencing to ensure their accuracy and integrity.

To construct infectious clones for different ALV subgroups, the RCASBP(A) infectious clone was utilized as the backbone, in which the gp85 gene was replaced with that of either ALV-B or ALV-J. Similarly, for generating infectious clones carrying the K282R mutation across various ALV subgroups, the corresponding subgroup-specific infectious clone served as the backbone, and a lysine-to-arginine substitution was introduced at the specified position.

### Antibodies and reagents

The monoclonal antibodies utilized in this study included anti-Flag M2 (Sigma-Aldrich, F1804 or CST, 14793S), anti-HA (Sigma-Aldrich, H9658 and H6908), anti-β-actin (Sigma-Aldrich, A1978), anti-Myc (Sigma-Aldrich, M4439 and C3956), anti-MARCH2 in rabbit (Solarbio, K007245P), anti-MARCH2 in mouse (stored in our laboratory), anti-ALV-A gp85 (stored in our laboratory), anti-ALV-B gp85 (stored in our laboratory), anti-ALV-J gp85 (stored in our laboratory), and anti-p27 (50). For secondary detection, we employed the goat anti-mouse IgG (H + L) cross-adsorbed secondary antibody, Alexa Fluor 546 conjugate (Invitrogen, A-11003), goat anti-rabbit IgG (H + L) cross-adsorbed secondary antibody, Alexa Fluor 488 conjugate (Invitrogen, A-11008), IRDye 800CW goat anti-mouse IgG secondary antibody (LI-COR, 926-32210), and IRDye 680RD goat anti-rabbit IgG secondary antibody (LI-COR, 926-68071), sourced from Invitrogen and LI-COR, as indicated. The following reagents were also used: bafilomycin A1 (MedChemExpress, HY-100558), chloroquine phosphate (MedChemExpress, HY-17589), and MG132 (MedChemExpress, HY-13259).

### RNA sequencing

The DF-1 cells were infected with ALV-A at an MOI of 0.1. At 120 h post-infection, the cells were collected for total RNA extraction using RNAiso Plus (TaKaRa, 9109), according to the manufacturer’s protocol. The RNA quality and integrity were assessed using a NanoDrop spectrophotometer (Thermo Fisher Scientific). The mRNAs with poly(A) tails were enriched and purified from the total mRNA pool using magnetic beads coated with poly(T) oligos. RNA-seq libraries were purified using the AMPure XP system and quantified using an Agilent High-Sensitivity DNA Assay on a Bioanalyzer 2100 system (Agilent). Libraries were sequenced using the NovaSeq 6000 platform (Illumina). Trimmomatic (v0.36) was used to preprocess the sequencing data and obtain high-quality sequences (clean data) for analysis. Reads were aligned to the reference genome using HISAT2 (v2.2.1). Differential gene expression was analyzed using edgeR (v3.26.6), applying the criteria of a |log_2_fold change| > 1 and FDR < 0.05. Genes were annotated using terms from the gene ontology (GO) database to determine the number of differentially enriched genes associated with each term. We then conducted a GO enrichment analysis using topGO based on the differentially expressed host genes in ALV-A-infected DF-1 cells compared with levels in non-infected cells at 120 h post-infection.

### ALV infection

DF-1 cells were seeded into 12-well tissue culture plates and subsequently infected with viruses that had been diluted to the appropriate titers. Following a 1.5-h adsorption period at 38.5°C, the cells were rinsed with DMEM devoid of FBS and then cultured in DMEM supplemented with 2% FBS for various durations.

### Generation of MARCH2-overexpression DF-1 cell line

A DF-1 cell line overexpressing MARCH2 was successfully established using a lentiviral delivery approach (51). To achieve this, the plasmid pLVX-IRES-zsmCherry-MARCH2-Flag was co-transfected with the packaging vectors psPAX2 and pVSV-G into HEK293T cells. Transfection was performed using PolyJet (Signagen, SL100688) as a transfection reagent to produce the lentivirus. Subsequently, the lentivirus was used to infect DF-1 cells, resulting in DF-1 cells with elevated MARCH2 expression. The overexpression of MARCH2 in these cells was validated via western blotting analysis using MARCH2 antibodies.

### Generation of MARCH2KO DF-1 cell line

DF-1-MARCH2KO cell lines were established using CRISPR/Cas9-mediated genome editing (52). Guide (g)RNA target sequences (gagcaggcggccgttcttgg) were designed using the E-CRISPR online tool (http://www.e-crisp.org). Synthetic DNA constructs, encompassing the U6 promoter, specific gRNA sequence, and gRNA scaffold, were amalgamated by performing overlapping PCR and were subsequently cloned into the pMD-18T vector (TaKaRa, 6011). DF-1 cells were transfected with a plasmid of gRNA and pMJ920, employing the TransIT-X2 transfection reagent, according to the manufacturer’s protocol. The cells exhibiting green fluorescence were subjected to flow cytometric sorting in 96-well plates, and monoclonal populations were ascertained through sequencing analysis and western blotting using an anti-MARCH2 antibody.

### Re-expression of the *MARCH2* gene in the MARCH2KO cell line

As previously described, a DF-1-MARCH2KO cell line re-expressing *MARCH2* was successfully established via a lentiviral delivery method. Specifically, lentiviral particles carrying the *MARCH2* gene were used to infect MARCH2KO cells, thereby facilitating the re-expression of *MARCH2* in these cells.

### Cell viability assay

The cells (4 × 10^4^ DF-1; WT or MARCH2KO) were seeded into 96-well plates. After culturing them for 6–8 h, 10 µL of CCK-8 solution from the CCK-8 kit (Dojindo, CK04) was added to the wells, according to the manufacturer’s instructions, to assess cell viability. After a 4-h incubation, the absorbance of the cells was measured at 450 nm.

### Transfection

DF-1 cells were seeded into 12-well culture plates and transfected with various plasmid constructs using the TransIT-X2 Dynamic Delivery System (Mirusbio, MIR 6000). After transfection, the cells were incubated for 24 h under standard culture conditions. Subsequently, the cells were harvested for RT-qPCR or western blotting analysis to assess gene expression and protein levels, respectively.

### RT-qPCR

Total RNA was isolated from distinct experimental groups using the RNAiso Plus reagent. Following extraction, RNA was reverse-transcribed into complementary DNA using a PrimeScript RT reagent kit (Vazyme, R223-01) that incorporates gDNA Eraser technology. Relative expression levels of the genes of interest were quantified using RT-qPCR. In this study, the RT-qPCR amplification was performed using the SYBR Green qPCR Kit (TOYOBO, QPS-201) under the following conditions: an initial denaturation at 95°C for 2 min, followed by 40 cycles consisting of 95°C for 5 s, 60°C for 30 s, and a melt curve analysis. The data were analyzed using the 2^−ΔΔC*t*^ method. The specific primers used in this research are available upon request.

Primer sequences are as follows: *p27* (F: 5′-GCAGCGAGATGCGAAGAT-3′, R: 5′-CCGCCAGGGAAGGATACA-3′), *GAPDH* (F: 5′-CCCCAATGTCTCTGTTGTTGAC-3′, R: 5′-CAGCCTTCACTACCCTCTTGAT-3′), *β-actin* (F: 5′-CAACACAGTGCTGTCTGGTGGTA-3′, R: 5′-ATCGTACTCCTGCTTGCTGATCC-3′), and *MARCH2* (F: 5′-gtacatcacccaggtcaccg-3′, R: 5′-ccctccccatttccaccttc-3′).

### Co-IP and western blotting

HEK293T cells were co-transfected with distinct plasmid constructs encoding FLAG, HA, or Myc epitope tags. At 36 h post-transfection, the cells were rinsed three times with PBS and lysed in 400 µL of western blotting and immunoprecipitation lysis buffer (Beyotime, P0013) for 20 min. The resulting cell lysates were centrifuged to pellet the debris, and the supernatants were incubated with monoclonal antibodies for 6–8 h to facilitate antigen–antibody complex formation. Subsequently, 40 µL of protein A/G agarose (Abmart, A10001) was introduced to the lysate mixture and incubated for an additional 6–8 h to enhance immunocomplex capture. The mixture was then centrifuged, and the pellet was washed five times with ice-cold PBS for 5 min at 4°C to remove unbound proteins.

For co-IP between gp85 and MARCH2 during ALV infection, DF-1 cells and DF-1-MARCH2KO cells were infected with ALV-A at an MOI of 0.1. At 48 h post-infection, the cells were washed three times with PBS and lysed in 200 µL of western blotting and immunoprecipitation lysis buffer for 20 min. The resulting lysates were then subjected to immunoprecipitation using a mouse-derived anti-MARCH2 antibody, following the procedure described previously herein.

For western blotting analysis, the collected samples were denatured in 5 × SDS loading buffer (Beyotime, P0015L) for 10 min, resolved on 12.5% SDS–polyacrylamide gels, and electrophoretically transferred onto nitrocellulose membranes. The membranes were then incubated with primary antibodies and horseradish peroxidase-conjugated secondary antibodies. The protein bands were detected and quantified using an Odyssey Infrared Imaging System (LICOR BioSciences, Lincoln, USA) for subsequent analysis.

### Pull-down assay

For the *in vitro* binding assay, recombinant human IgG-Fc-tagged ALV-A gp85 proteins and His-tagged MARCH2 proteins were expressed in 293T cells, respectively. Then the cell culture lysate was harvested, and the respective proteins were purified using protein A agarose or Ni-NTA agarose columns. The Ni-NTA agarose column was subsequently incubated with 20 µg of His-tagged MARCH2 together with Fc-tagged ALV-A gp85 at 4°C for 2 h with gentle agitation. After five washes with ice-cold PBS, the bound proteins were separated by SDS-PAGE and detected by western blotting.

### Confocal microscopy

DF-1 cells, cultured in 35 mm tissue culture dishes (Biosharp, BS-20-GJM), were co-transfected with the specified plasmid constructs or infected with ALV-A. Following a 24-h incubation period, the cells were rinsed three times with PBS and subsequently fixed in a 4% (vol/vol) paraformaldehyde solution (Biosharp, BL539A) for 30 min. After fixation, the cells were permeabilized and incubated with the appropriate primary monoclonal antibodies, followed by incubation with the corresponding set of secondary antibodies for detection. Subsequently, the cells were counterstained with 4′,6-diamidino-2-phenylindole for 10 min to visualize the nuclear DNA. The stained cells were examined using a confocal laser scanning microscope (LSM980, Zeiss, Germany) for the high-resolution imaging of cellular structures and antigen localization.

### Virus growth curves

DF-1 cells were cultured in flasks and subsequently infected with ALV-A at an MOI of 0.1. Supernatants from each experimental group were collected at 48 and 120 h post-infection. Viral titers in the supernatants were quantified by performing a TCID_50_ assay. Based on the TCID_50_ data, a viral growth curve was constructed to graphically represent the replication dynamics of the viruses over time.

### Assessment of the role of MARCH2 at different stages of the ALV replication cycle

Effect of MARCH2 on ALV entry into host cells (53): DF-1 cells were seeded in 12-well plates and cultured for 24 h. The cells were then transfected with either a MARCH2 expression plasmid or an empty vector control. After 24 h of transfection, both the cells and ALV were pre-chilled on ice for 30 min. The chilled virus was subsequently applied to the pre-cooled cells and incubated at 4°C for 2 h. Following viral adsorption, the cells were transferred to a 37 °C incubator and cultured for an additional 24 h. Cell pellets were collected, and genomic DNA was extracted. ALV-specific primers were used for qPCR analysis to evaluate the effect of MARCH2 overexpression on viral entry.

Effect of MARCH2 on ALV reverse transcription (54): DF-1 cells were plated in 12-well plates and cultured for 24 h. They were then transfected with either the MARCH2 expression plasmid or the empty vector control. At 24 h post-transfection, ALV was inoculated into the cells. At 8 h post-infection (the time point at which reverse transcription is completed), cell pellets were harvested. Genomic DNA was extracted and subjected to qPCR analysis using ALV-specific primers to assess the effect of MARCH2 on the reverse transcription process.

Effect of MARCH2 on ALV integration (54): DF-1 cells were seeded in 12-well plates and cultured for 24 h before transfection with either the MARCH2 expression plasmid or the empty vector control. At 24 h post-transfection, ALV was inoculated. The cells were harvested at 24 h post-infection (the time point marking the completion of viral integration). The genomic DNA of infected DF-1 was extracted and separated via agarose gel electrophoresis. The band corresponding to high-molecular-weight genomic DNA was excised and purified. The purified DNA was then analyzed via qPCR using ALV-specific primers to determine the effect of MARCH2 on viral integration.

Effect of MARCH2 on ALV promoter activity (18): DF-1 cells were plated in 12-well plates and cultured for 24 h. The pGL3-ALV-LTR plasmid, which contains the ALV long terminal repeat (LTR) driving luciferase expression, was co-transfected with either the MARCH2 expression plasmid or the empty vector control. At 24 h post-transfection, luciferase activity was measured using a luciferase reporter assay system to evaluate the effect of MARCH2 overexpression on ALV promoter activity.

Effect of MARCH2 on ALV protein expression and virion release (55, 56): DF-1 cells were seeded in 12-well plates and cultured for 24 h. An infectious clone plasmid of ALV-A was co-transfected with either the MARCH2 expression plasmid or the empty vector control. At 48 h post-transfection, cell culture supernatants and cell lysates were collected. Western blotting analysis was performed using specific monoclonal antibodies against ALV p27 and gp85 proteins to assess the levels of viral protein expression and particle release.

### Rescue of recombinant virus

Using the TransIT-X2 Dynamic Delivery System, WT and mutant infectious clone plasmids of ALV were independently transfected into DF-1 cells. Seven days post-transfection, the culture supernatants containing viral stocks were harvested and subsequently subjected to blind passage onto fresh DF-1 cells. The titers of the rescued recombinant viruses were determined based on TCID_50_ values using the Reed and Muench method.

### Model drawing

The model by which MARCH2 suppresses ALV replication by targeting gp85 for degradation was created using FigDraw. The authorization code for this model is RPTRAe6420.

### Statistical analysis

The experiments were performed in triplicate, with each result representative of three separate experimental trials. Data are expressed as the mean ± standard deviation. Statistical analyses were conducted using GraphPad Prism software (version 7.03; GraphPad Software, San Diego, CA, USA). Differences between groups were evaluated using the Student’s *t*-test. The threshold for statistical significance was set at *P* < 0.05.

## Data Availability

Data supporting the findings of this study are available on request from the corresponding author.

## SUPPLEMENTAL MATERIAL

Fig. S1 jvi.01616-25-s0001.tif Identification of the MARCH2-overexpressing cell line via western blotting.

Fig. S2 jvi.01616-25-s0002.tif Validation of the interaction of ALV-A-gp85 (with an Fc tag) with MARCH2 (with a Flag tag) as assessed by pulldown assays.

Table S1 jvi.01616-25-s0003.xlsx Relative expression of chicken genes upon ALV-A infection at 120 hpi.

Table S2 jvi.01616-25-s0004.xlsx RNA-seq analysis of MARCH expression levels upon ALV-A infection compared to those without infection in DF-1 cells at 120 hpi.

ASM does not own the copyrights to Supplemental Material that may be linked to, or accessed through, an article. The authors have granted ASM a non-exclusive, world-wide license to publish the Supplemental Material files. Please contact the corresponding author directly for reuse.
