# Supplementary figures and images for: MARCH2 inhibits avian leukosis virus replication by targeting gp85 for ubiquitination and degradation
Source: J Virol. 2025 Dec 3;99(12):e01616-25. doi: 10.1128/jvi.01616-25 (PMC12724284; doi:10.1128/jvi.01616-25)

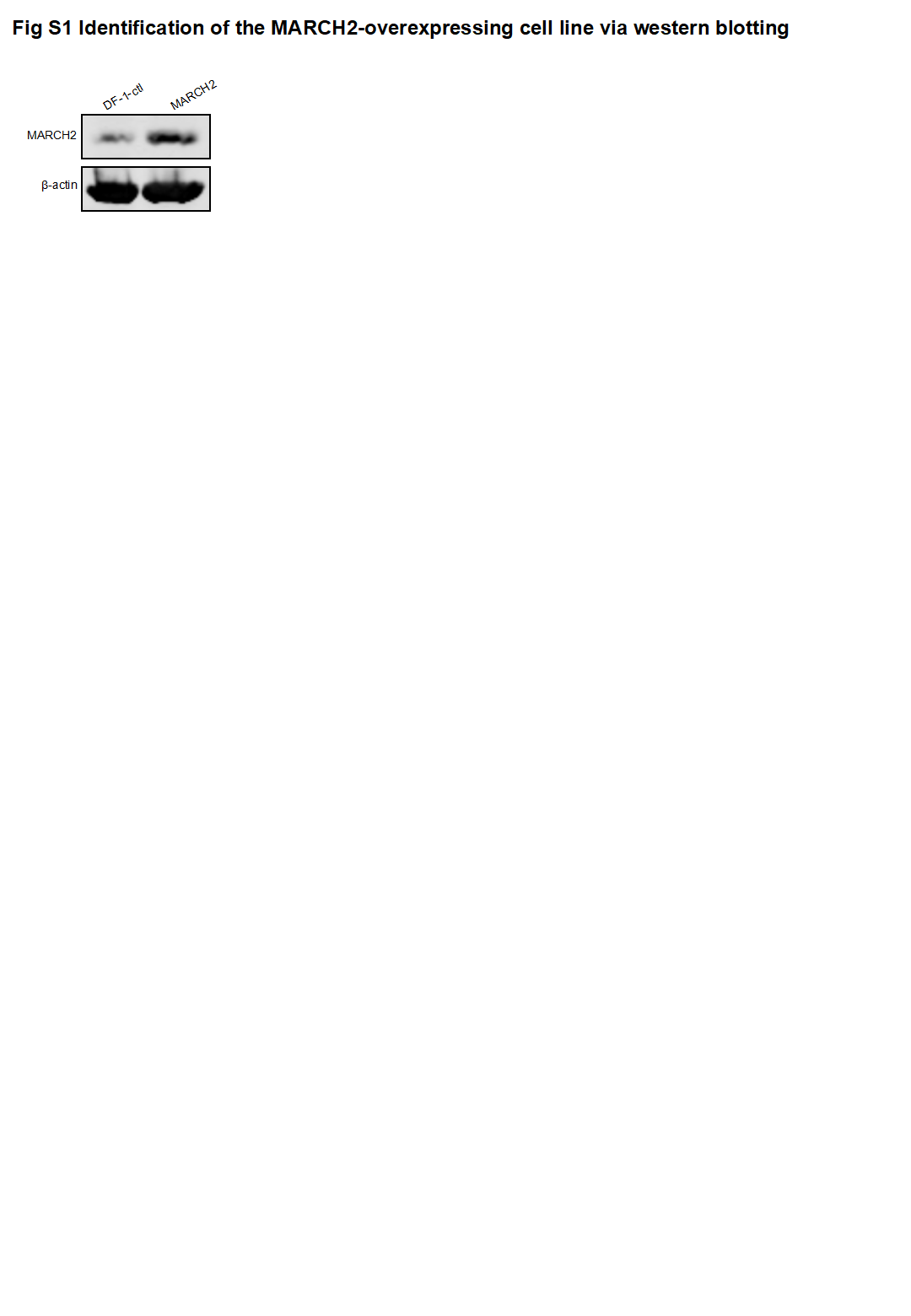

Supplement: Fig. S1 — Identification of the MARCH2-overexpressing cell line via western blotting. [file jvi.01616-25-s0001.tif]

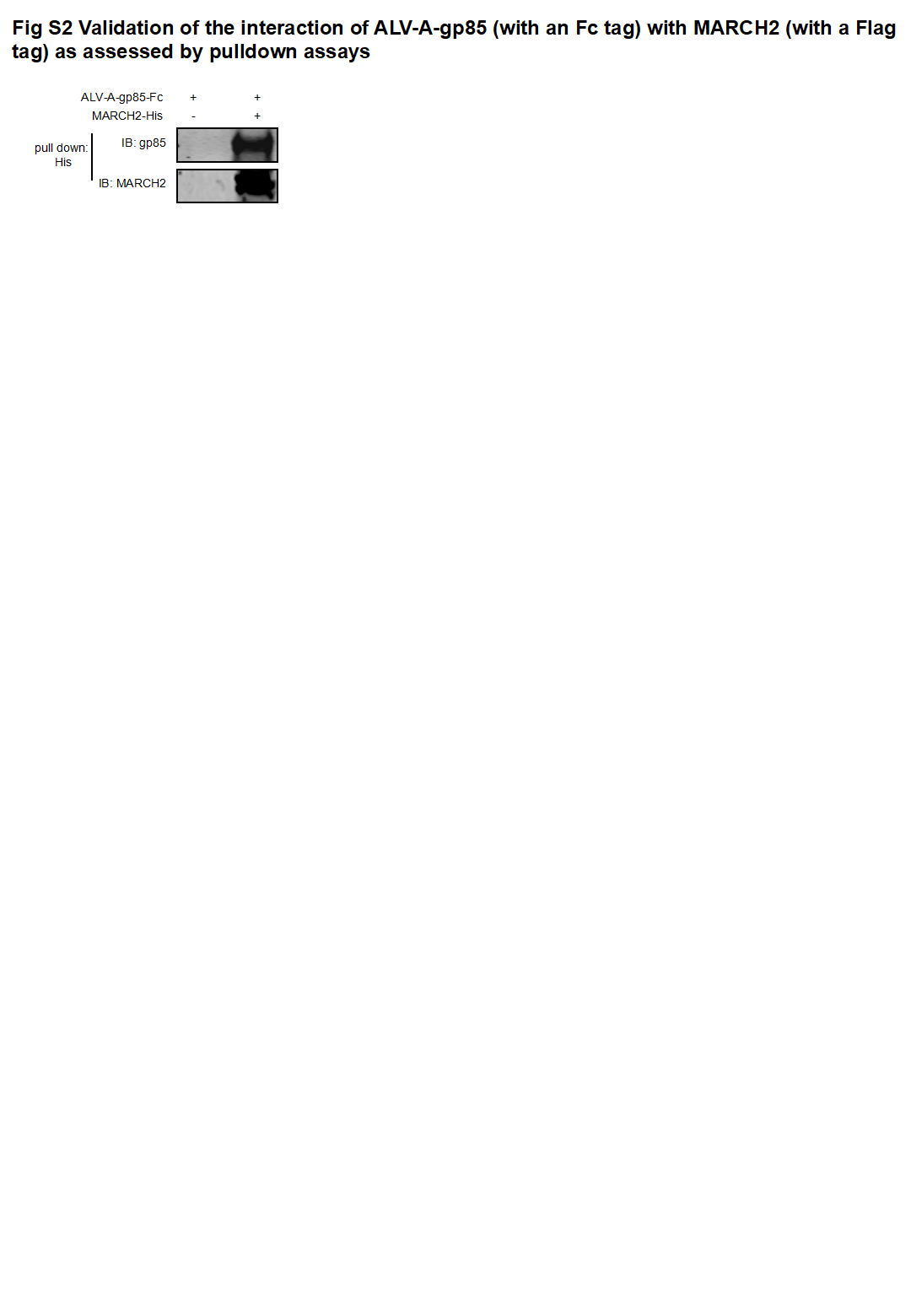

Supplement: Fig. S2 — Validation of the interaction of ALV-A-gp85 (with an Fc tag) with MARCH2 (with a Flag tag) as assessed by pulldown assays. [file jvi.01616-25-s0002.tif]
